# Radiological and Clinical Outcomes After Navigated Tubular Unilateral Laminotomy for Bilateral Decompression (ULBD) for Lumbar Spinal Stenosis Among Patients with Concurrent Degenerative Scoliosis: A Short-Term Retrospective Case Series

**DOI:** 10.3390/brainsci16020183

**Published:** 2026-02-01

**Authors:** Mateusz Bielecki, Chibuikem A. Ikwuegbuenyi, Yizhou Xie, Jessica Berger, Catherine Mykolajtchuk, Anne Schlumprecht, Rodolfo Villalobos-Diaz, Noah Willett, Mousa K. Hamad, Galal Elsayed, Ibrahim Hussain, Osama N. Kashlan, Roger Härtl

**Affiliations:** Department of Neurological Surgery, Och Spine at New York Presbyterian Hospital, Weill Cornell Medicine, New York, NY 10021, USA; mb.spine1@gmail.com (M.B.);

**Keywords:** ULBD, unilateral laminotomy for bilateral decompression, minimally invasive spine surgery, MISS, degenerative lumbar scoliosis, lordosis, navigation

## Abstract

**Background/Objectives:** Adult degenerative scoliosis (ADS) is a spinal disease causing pain and reduced mobility, often occurring with degenerative lumbar spinal stenosis (DLSS). While fusion stabilizes the spine, it has drawbacks like loss of motion and adjacent segment degeneration. Minimally invasive techniques, such as tubular unilateral laminotomy for bilateral decompression (tULBD), provide a less invasive alternative, but their impact on ADS with DLSS is underexplored. This study examines the short-term effects of navigated tULBD on radiological and clinical outcomes in this patient population. **Methods:** This retrospective single-center study analyzed patients aged ≥18 years with DLSS and ADS (Cobb angle ≥ 10°), with or without grade I spondylolisthesis, who underwent navigated tULBD between June 2019 and October 2022. Radiological parameters were assessed pre- and post-operatively using AI-powered FXA™ Version 1.33, Raylytic Software GmbH, Leipzig, Germany, while clinical outcomes were evaluated using the Oswestry Disability Index (ODI) and Numeric Rating Scale (NRS) for back and leg pain. Statistical analyses were conducted with R Studio. **Results:** This study included 20 patients (mean age 74.6 ± 7.6 years, body mass index [BMI] 26.08 ± 3.7 kg/m^2^), with a median follow-up of 2 months. Most underwent single-level decompression (45%), with a median of 2 surgical levels (IQR: 1–3). Radiological parameters showed no significant changes (*p* > 0.05). Clinically, the median NRS back improved from 5 (IQR: 3–9) preoperatively to 2 (IQR: 0–2) postoperatively (*p* = 0.009) and 1 (IQR: 0–4.5) at follow-up (*p* = 0.004). NRS leg scores dropped from 3.5 (IQR: 0–5) to 0 postoperatively and at follow-up (*p* = 0.02, *p* = 0.04). ODI improved from 37.8 (IQR: 29–42.5) preoperatively to 17.5 (IQR: 5–24) at follow-up (*p* = 0.04). There were no neurological complications. **Conclusions:** Navigated tULBD is a promising, minimally invasive option for mild ADS and DLSS. It provides significant pain and disability relief without adversely affecting stability and alignment. Long-term studies are needed to confirm durability and efficacy, particularly in severe cases.

## 1. Introduction

Adult Degenerative Scoliosis (ADS) is a spinal deformity that develops due to age-related degeneration, leading to pain and reduced mobility [1,2]. ADS has been identified radiographically in up to 68% of asymptomatic elderly individuals. Many patients with ADS also develop degenerative lumbar spinal stenosis (DLSS) [3], spinal canal narrowing compresses neural elements, exacerbating pain and limiting mobility [4,5]. The combination of these conditions complicates treatment, as both ADS and DLSS are conditions that could be addressed surgically.

Conservative treatment options for mild ADS and DLSS include observation, physical therapy, and selective use of bracing, though the latter is generally not effective for neurogenic symptoms [2,5,6]. When conservative measures fail, surgical intervention may be considered. In some cases, particularly those with progressive deformity, instability, or significant sagittal imbalance. Fusion-based approaches are used to restore alignment and stabilize the spine [4,7]. However, fusion is associated with loss of segmental motion and risks such as adjacent segment degeneration, especially in elderly patients or those with only mild deformity. These considerations have prompted growing interest in motion-preserving surgical alternatives for well-selected patients with ADS and DLSS [8].

Advances in surgical technologies, such as 2D/3D imaging systems, tubular retractors, and microscopes, have driven minimally invasive spine surgery (MISS). One example is the tubular unilateral approach for bilateral decompression (tULBD), a technique for treating spinal stenosis. This technique, also known as the “over-the-top” approach, has been previously described by Kirnaz et al. and allows for bilateral decompression and foraminotomy through a unilateral approach [9]. The key advantages of this technique include minimizing iatrogenic destabilization while still providing adequate decompression [10]. Moreover, the procedure preserves spinal mobility by avoiding fusion. This allows patients to retain their range of motion and flexibility, which is crucial for their overall quality of life [11].

In patients with DLSS and mild ADS—typically defined by Cobb angles between 10° and 20°, without significant sagittal imbalance or instability—the clinical dilemma often centers on whether to perform decompression alone or to include spinal fusion [6,12,13]. Decompression alone may be appropriate when symptoms are primarily driven by neural compression rather than structural deformity [14,15]. Surgeons may favor this approach in older or medically frail patients, where the risks of fusion—including greater blood loss, longer operative times, and higher complication rates—may outweigh potential benefits [5,16,17]. However, traditional decompression techniques risk destabilizing the spine, particularly in the setting of pre-existing deformity [15,18]. tULBD offers a minimally invasive solution. This study evaluates the short-term impact of this approach on spinal alignment and clinical outcomes in this patient population.

## 2. Materials and Methods

### 2.1. Study Design and Clinical Setting

This retrospective study was conducted at a single tertiary medical center. It included patients who underwent the tULBD procedure for DLSS and ADS by a single surgeon between June 2019 and October 2022. A thorough retrospective analysis of patient radiographs was performed to assess the presence of scoliosis. The inclusion criteria were individuals aged 18 or older who had undergone tULBD for symptomatic DLSS (presenting with neurogenic claudication and/or radiculopathy refractory to conservative treatment), with or without coexisting Meyerding grade I spondylolisthesis, and radiographic evidence of ADS (global Cobb angle ≥ 10°). Exclusion criteria included patients younger than 18, those with spinal surgery other than tULBD, and individuals with grade II-IV spondylolisthesis, spondyloptosis, or mild spinal curvature (global Cobb angle < 10°).

### 2.2. Surgical Procedure

**Figure 1:**
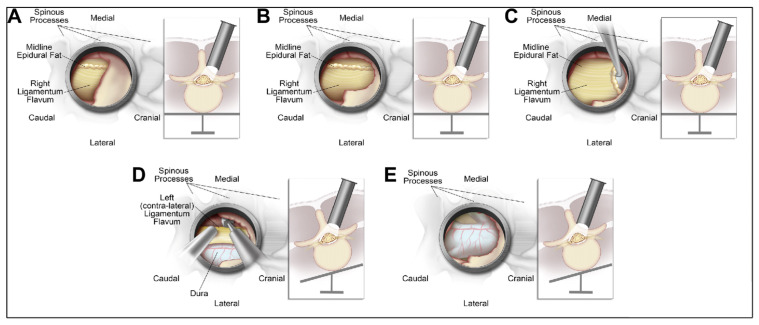
Key technical steps of the navigated tubular unilateral laminotomy for bilateral decompression (tULBD) procedure as was performed in this study. (**A**) Tubular placement for a right-sided approach. Under the microscope, the inferior edge of the lamina and the inferior edge and base of the spinous process are exposed. The union of these 2 bony structures serves as a landmark for the starting point of the lumbar decompression. (**B**) The laminotomy is conducted using a 3-mm curved drill and bayoneted 2- and 3-mm Kerrison rongeurs. The ligamentum flavum (LF) is exposed but not removed at this stage to protect the underlying dura and reduce the risk of cerebrospinal fluid (CSF) leak. (**C**) After ipsilateral laminotomy, ipsilateral flavectomy is performed. The LF is gently separated from the dura using a ball-tip instrument. Starting cranially, a Kerrison rongeur is used to remove the ipsilateral LF down to its caudal insertion at the lamina below. (**D**) Drilling of the contralateral lamina; the sucker is used to protect and gently depress the LF and underlying structures, while the fluted 3-mm matchstick drill is used to undercut the contralateral lamina and other bony structures to complete the decompression. This may require further tilting of the table and/or retractor to optimize access. (**E**) A complete removal of the LF is achieved, and the dura is safely exposed. The contralateral exiting and traversing nerve roots may also be exposed if necessary. Used with permission from Operative Neurosurgery, vol 13, issue 2, authors Boukebir, M.A., Berlin, C.D., Navarro-Ramirez, R., Heiland, T., Schöller, K., Rawanduzy, C., Kirnaz, S., Jada, A., Härtl, R., pp. 232–245, copyright 2017, with permission from Wolters Kluwer [10].

Surgery was performed by an experienced surgeon with extensive experience in tULBD [10,19,20]. Surgeries were performed under general anesthesia, with the patient in a prone position on a Wilson frame. Total navigation guidance is utilized throughout the surgery to ensure accurate localization of the target level. The intraoperative navigation system (Brainlab AG, Feldkirchen, Germany) is set up, and a reference array is securely positioned on the contralateral iliac crest. An intraoperative CT scan is performed to acquire the image for navigation. Three-dimensional navigation is utilized throughout the surgery to ensure accurate localization of the target level. A small skin incision, 1.5–2 cm lateral to the midline, is made, and the fascia is opened longitudinally. The first dilator is inserted at a slightly medial angle to identify the spinous process and lamina base, with navigation confirming its correct positioning. Sequential dilation is performed until the appropriate dilation is reached, and the retractor is secured to the table-mounted arm. Under the surgical microscope, the starting point for drilling is exposed at the intersection of the base of the spinous process and the medial lamina. A laminotomy is then carried out by drilling in a caudal-to-cranial direction, approaching the insertion of the ligamentum flavum. The remaining lamina portion is removed using Kerrison rongeurs, then carefully separating the ligamentum flavum from the dura mater using a ball-tip instrument. To access the contralateral side, the operating table is angled away from the surgeon (10–20°), and the tubular retractor is angled more medially. The interface between the contralateral ligamentum flavum and the covering bone is identified, and laminotomy and flavectomy are performed using the same technique. Adequate decompression of the contralateral foramen is confirmed using intraoperative imaging. Finally, the table is returned to a neutral position, and the tubular retractor is angled laterally to the original position to inspect the ipsilateral lateral recess for adequate decompression. The tube is then removed, and the incision is closed. Figure 1 shows the steps in microtubular decompression.

### 2.3. Radiological Assessment

**Figure 2:**
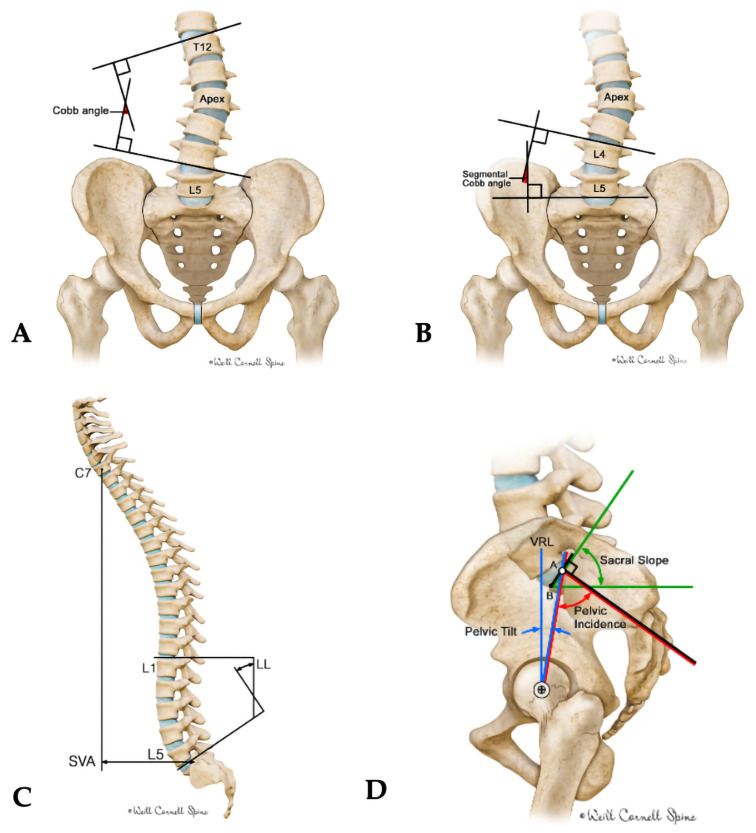
Illustration depicting measurements of spinal alignment and curvature. (**A**) Global Cobb angle: Angle between superior endplate of the uppermost tilted vertebra and inferior endplate of the lowest tilted vertebra. (**B**) Segmental Cobb angle: Same method applied to a specific spinal segment. (**C**) Sagittal vertical axis (SVA) and lumbar lordosis (LL): SVA is the horizontal distance from the C7 plumb line to S1; LL is the angle between the superior endplates of L1 and S1. (**D**) Pelvic parameters: Pelvic incidence (PI) is the angle between the sacral endplate and femoral head axis; pelvic tilt (PT) is the angle between the sacral midpoint-femoral head axis and vertical reference line (VRL); sacral slope (SS) is the angle between the sacral endplate and horizontal.

Spinal stability was assessed pre- and post-surgery using radiological measurements of spinopelvic parameters: pelvic incidence (PI), pelvic tilt (PT), sagittal slope (SS), and lumbar lordosis (LL). Global alignment measurements were also recorded, precisely the sagittal vertical axis (SVA) and coronal parameters, including the global Cobb angle (GCA) and segmental Cobb angle (SCA), Figure 2A–D. All these measurements were conducted using the FXA™ Version 1.33, Raylytic Software GmbH, Leipzig, Germany developed by Raylytic AI GmbH in Esslingen, Germany, an AI-powered tool. This tool has been validated in previous studies, including assessments of accuracy and excellent inter- and intra-observer reliability using intraclass correlation coefficients across multiple spinal alignment parameters [21,22].

### 2.4. Clinical Outcome

Patients completed validated outcome questionnaires, including the oswestry disability index (ODI) [23], and a numeric rating scale (NRS) for leg and back pain before surgery and at follow-up visits. Routine neurological examinations were performed preoperatively and at follow-up as part of standard clinical care, but findings were not systematically recorded for analysis. We also collected patient demographic information, such as age, body mass index (BMI), and procedural details, including surgical level, number of levels operated, estimated blood loss, procedure duration, and hospital length of stay, from the electronic medical records.

### 2.5. Statistical Analysis

Continuous data were described using either mean (standard deviation) or median with interquartile range (IQR), while count data were presented as counts and percentages (%). We assessed the normality of the data using the Shapiro–Wilk test. To compare measurements at different time points, we employed either the Wilcoxon Signed Rank Test or, when applicable, the paired t-test. Statistical Significance was set at 0.05, and all statistical analyses were conducted using R Studio version 4.1.2 (Vienna, Austria). Visualizations were performed using GraphPad Prism version 10.4.1 (San Diego, CA, USA).

## 3. Results

Table 1 shows the sociodemographic, clinical characteristics, hospitalization, and operative information of the 20 patients in the study. The mean age of the patients was 74.6 ± 7.6 years, with 12 males and eight females, and a mean BMI of 26.08 ± 3.7 kg/m^2^. Thirteen patients presented with weakness. Most patients had a 1-level decompression (45%), with L3/4 level being the most decompressed (38.5%), followed by L4/5 (25.6%). The average surgical time was 130.1 ± 52.7 min, with an average blood loss of 43.3 ± 14.2 mL.

The radiological outcomes are presented in Table 2. All parameters had no statistically significant differences between pre-operative and post-operative values. Pelvic incidence slightly decreased from a median of 54.8° (IQR 46.7–62.3°) to 53.8° (IQR 47.3–62.3°) (*p* = 0.07). Pelvic tilt also decreased from 28.9° (22–30.9°) to 26.3° (22–32.1°) (*p* = 0.16), while sacral slope remained stable at 31.4° (24.9–33.3°) vs. 29.4° (26.3–33.8°) (*p* = 0.70). No significant change was seen in lumbar lordosis (42.8° ± 15.5° to 42.4° ± 14.8°, *p* = 0.75), sagittal vertical axis (34.4 mm to 36.2 mm, *p* = 0.53), global Cobb angle (18.6° to 19.6°, *p* = 0.08), or segmental Cobb angle (6.0° to 5.7°, *p* = 0.53).

**Figure 3:**
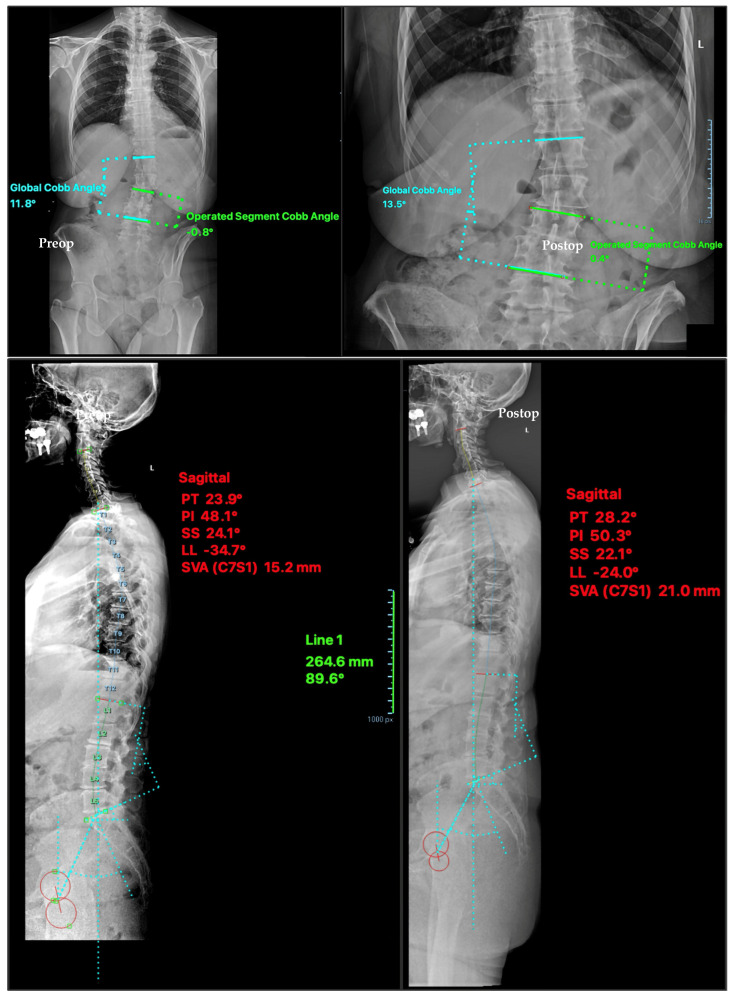
Representative Case: Preoperative and Postoperative Imaging Evaluation. Preoperative (**left**) and postoperative (**right**) radiographs of a representative case demonstrating changes in spinal alignment following navigated tubular unilateral laminotomy for bilateral decompression (tULBD). The top panel (AP view) shows changes in global and operated segment Cobb angles. The bottom panel (sagittal view) illustrates modifications in sagittal alignment parameters, including pelvic tilt (PT), pelvic incidence (PI), sacral slope (SS), lumbar lordosis (LL), and sagittal vertical axis (SVA). No substantial changes in alignment were observed following surgery.

Figure 3 shows a representative case with radiological measurements performed in this study.

### 3.1. Clinical Outcomes

#### 3.1.1. NRS Back

**Figure 4:**
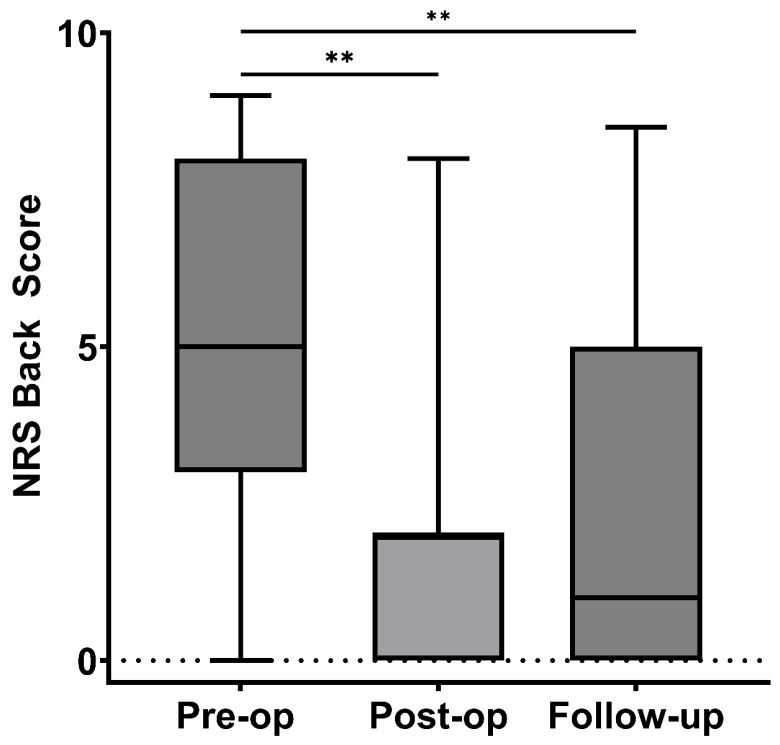
NRS back (Numeric Rating Scale) Scores at Pre-op, Post-op, and Follow-up. Box plot showing NRS back scores at pre-operative, post-operative, and follow-up time points—missing values: Pre-op (1), post-op (3), Follow-up (5), ** *p* ≤ 0.01.

The Back NRS scores improved significantly over time. The median score decreased from 5 (IQR 3–9) preoperatively to 2 (IQR 0–2) immediately postoperatively (*p* = 0.009), as shown in Figure 4. At the last follow-up, the median score declined to 1 (IQR 0–4.5) (*p* = 0.004), reflecting sustained pain relief compared to the preoperative period. However, there was no statistically significant change between the immediate postoperative and follow-up periods (*p* = 0.40).

#### 3.1.2. NRS Leg

**Figure 5:**
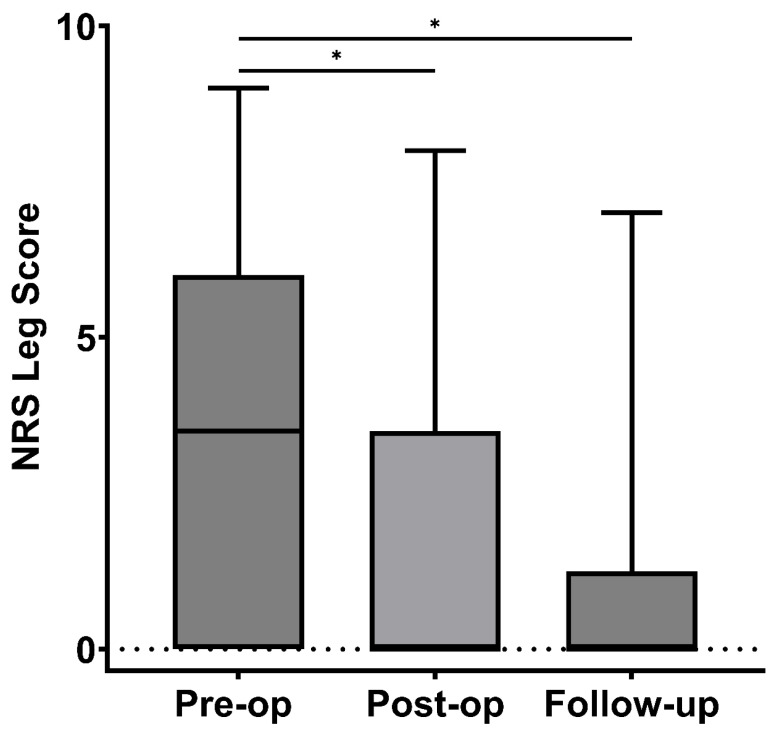
NRS Leg (Numeric Rating Scale) Scores at Pre-op, Post-op, and Follow-up. Box plot illustrating NRS leg scores at three time points: pre-operative, post-operative, and follow-up. Missing values: Pre-op (3), post-op (6), Follow-up (6), * *p* ≤ 0.05.

NRS leg scores showed a substantial reduction following surgery and at follow-up. The median score was 3.5 (IQR 0–5) pre-operatively, dropping to 0 (IQR 0–3.1) post-operatively (*p* = 0.02) (Figure 5). This improvement persisted, with the NRS leg score remaining at 0 (IQR 0–0) at the last follow-up (*p* = 0.04), indicating sustained relief in leg pain. However, there was no statistically significant change between the immediate postoperative and follow-up periods (*p* = 0.61).

#### 3.1.3. ODI

**Figure 6:**
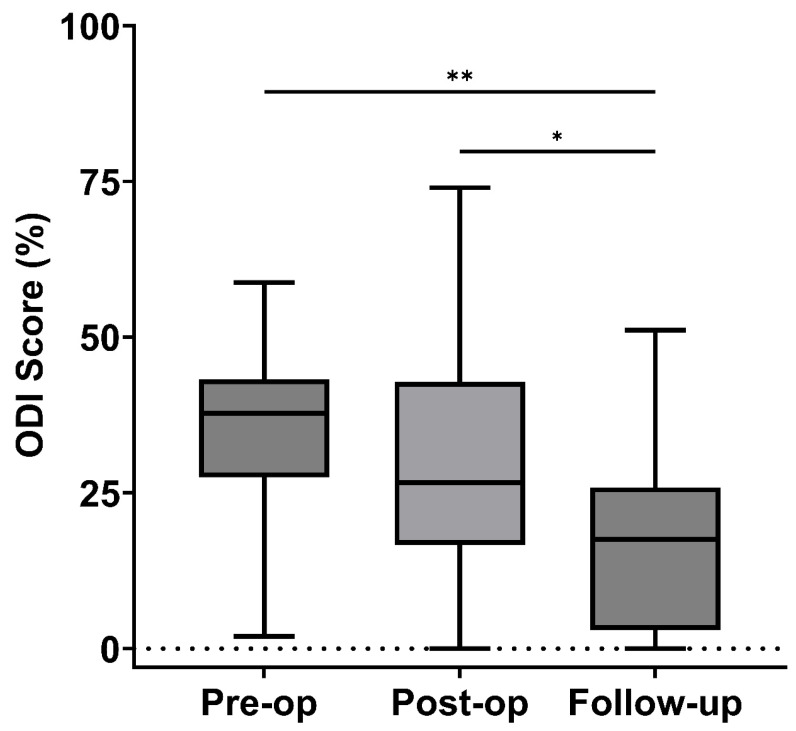
ODI (Oswestry Disability Index) Scores at Pre-op, Post-op, and Follow-up. Box plot depicting ODI scores at pre-operative, post-operative, and follow-up stages. Missing values: Pre-op (3), post-op (5), Follow-up (5), * *p* ≤ 0.05, ** *p* ≤ 0.01.

The ODI scores demonstrated improvement over time. Preoperatively, the median ODI was 37.8% (IQR 29–42.5). Postoperatively, it decreased to 26.7% (IQR 20–36.7), though this reduction was not statistically significant (*p* = 0.11). Between the immediate postoperative period and the follow-up, a significant improvement was observed (*p* = 0.04). By the last follow-up, the median ODI had further improved to 17.5% (IQR 5–24) (*p* = 0.004), reflecting a meaningful reduction in disability, as illustrated in Figure 6.

### 3.2. Perioperative Safety Outcomes

No intraoperative or postoperative neurological complications were observed in this cohort. There were no cases of cerebrospinal fluid leaks, surgical site infection, or perioperative medical complications. Additionally, no patient required early reoperation, revision decompression, or conversion to fusion during the follow-up period.

## 4. Discussion

ADS is characterized by the progressive degeneration of spinal motion segments, resulting in the development of a scoliotic curve [24,25]. Degeneration causes uneven facet joint loading, worsening asymmetry and intervertebral collapse [26,27]. The compounding nature of this process has led some surgeons to consider fusion surgery as a means to restore normal spinal alignment and biomechanical function [5,16,17]. However, the typical Cobb angle of the curve in ADS is relatively mild, usually ranging from 10 to 20° [8,9,10]. This modest curvature has raised questions about the necessity of fusion surgery for this condition [6,12,13]. As a result, the most appropriate therapeutic strategy for degenerative scoliosis remains a topic of debate.

Classification systems have been developed to address this question and attempt to standardize treatment [1,6,28]. The Scoliosis Research Society-Schwab classification, was established to categorize patients with ADS and provide treatment recommendations [29]. This system uses radiographic measures to diagnose scoliosis and guide surgery but is criticized for ignoring key clinical factors.

In response to these limitations, Fernando E. Silva and Lawrence G. Lenke [6] developed a new classification system incorporating clinical and radiographic parameters. The system classifies ADS into six levels, each with specific treatment strategies for better clinical guidance. Despite these advancements, subsequent literature has raised concerns about the recommended fusion levels in both classification systems [30,31,32]. Some researchers suggest that the extent of spinal fusion could be reduced further, highlighting the ongoing evolution of ADS treatment strategies. While the debate over classification systems and fusion levels continues, several studies have demonstrated that decompression alone could achieve both preferable clinical and radiographic outcomes in certain cases of ADS [14,15]. This research provides an alternative perspective to the fusion-centric approaches discussed earlier.

Kenji Masuda et al. conducted a retrospective analysis of 57 patients with degenerative lumbar disease, featuring Cobb angles ranging from 10° to 25°—the study compared clinical and radiological results after decompression alone versus decompression with fusion surgery [14]. Using preoperative and postoperative Cobb angles, LL, and Japanese Orthopedic Association (JOA) scores as evaluative indexes, the study demonstrated improved JOA scores and preserved Cobb angles after surgery for both techniques, with no statistically significant difference between the two approaches [14]. Similarly, Gadiya et al. retrospectively analyzed 51 ADS patients who underwent lumbar decompression (2006–2016) using Cobb angle, LL, visual analog scale (VAS), and ODI. The results revealed that lumbar decompression in degenerative scoliosis is associated with good functional outcomes and less curve progression at mid-term follow-up [15].

These studies suggest that decompression alone may be a viable option for some ADS patients, potentially offering similar benefits to more invasive fusion procedures while minimizing surgical complexity and associated risks. Traditionally, open surgical decompression for DLSS involved wide bilateral laminectomies and medial facet undercutting [18]. However, these approaches risk postoperative flexion instability by removing supraspinous and interspinous ligaments, potentially worsening spinal instability in ADS patients [15]. Advances in enabling technologies have improved MISS techniques [33]. MISS aims to achieve effective decompression while minimizing tissue damage and iatrogenic instability risk, offering promising alternatives for ADS treatment.

One such MISS technique is the tULBD, which offers advantages over traditional open methods, including reduced postoperative complications, less tissue manipulation, lower blood loss, decreased surgical site infection, and earlier ambulation and return to work [10,34]. A recent biomechanical study showed that tULBD produces significantly less instability than traditional midline laminectomy [35], crucial for ADS patients. Despite literature highlighting MISS’s advantages over open approaches [33,36,37,38], clinical outcomes of tULBD alone in ADS remain understudied. Our study explored whether tULBD could achieve satisfactory clinical and radiographic outcomes in ADS patients with concurrent spinal stenosis. The results align with the existing literature [14,15,39,40].

The analysis of radiological outcomes revealed no significant changes in postoperative alignment parameters, including PI, PT, SS, LL, SVA, GCA, and SCA, compared to preoperative values. This aligns with expectations, as surgery targeted symptom relief, not structural correction, and avoided iatrogenic destabilization a common risk in traditional decompression. By utilizing a motion-preserving technique like tULBD, we aimed to preserve tension band and the contralateral facet joint, thereby maintaining the pre-existing spinal alignment. This approach avoids risks of complex deformity correction in older patients while ensuring that the scoliotic curve does not acutely progress due to surgical intervention. Consistent with prior studies, decompression offers short-term pain relief without affecting curve progression or stability [15].

Postoperative NRS back scores significantly improved and continued through follow-up, showing sustained pain relief. NRS leg scores followed a similar trend. Though ODI showed no immediate change, disability decreased significantly by the final follow-up. This functional improvement is likely attributed to patient rehabilitation and increased daily activities, leading to enhanced overall outcomes.

This small retrospective study provides preliminary evidence that MISS decompression alone may significantly alleviate the clinical symptoms caused by DLSS without affecting the radiological parameters of spine alignment. Thus, avoiding the complications associated with more extensive surgical approaches common for patients with ADS. These preliminary findings, support the philosophy of “achieving more by doing less” [15]. These findings contrast with Asada et al., who reported worse functional outcomes in patients with spinal curvature over 20° after MISS decompression alone [41]. The authors emphasized careful surgical planning, especially if it involves decompression of the levels between or across the end vertebrae of the Cobb angle. Proper patient selection and precise symptom level identification are crucial for good clinical outcome. As mentioned in the framework known as the “6 T’s of Minimally Invasive Spine Surgery” defined by Dr. Roger Härtl [42], only after the pathology is adequately targeted can the surgeon evaluate and select the optimal plan for the patient, especially in complex cases such as those involving ADS.

From a translational perspective, integrating navigated tULBD with AI-assisted radiographic analysis entails additional upfront costs related to navigation platforms and software infrastructure. These costs must be balanced against potential benefits, including reduced tissue disruption, preservation of spinal stability, avoidance of fusion in selected patients, and improved efficiency and reproducibility of radiographic assessment. Formal cost-effectiveness analyses were beyond the scope of this study, but future investigations incorporating economic endpoints will be important to define the value of AI-supported minimally invasive decompression strategies.

Endoscopic decompression techniques have also been proposed as motion-preserving alternatives for the treatment of lumbar spinal stenosis, including in selected patients with mild degenerative scoliosis [43]. Direct comparative evaluation between navigated tULBD and endoscopic approaches was beyond the scope of this study. Differences in visualization, instrumentation, learning curve, and reliance on navigation or fluoroscopy may influence patient selection and outcomes, underscoring the need for future comparative or registry-based studies.

Although this study provides valuable evidence supporting the safety and short-term effectiveness of navigated tULBD in patients with DLSS and mild ADS, it is limited by its retrospective design, which introduces potential bias. The single center setting and small sample size restrict generalizability, and no a priori sample size or power calculation was performed, as this analysis included all eligible patients treated during the study period; accordingly, these findings should be interpreted as exploratory, highlighting the need for larger, multicenter studies. The short follow-up duration (median two months) substantially limits the ability to assess the durability of clinical improvement, long-term spinal alignment stability, and the potential for delayed complications, including curve progression, adjacent segment degeneration, or the need for fusion or reoperation. In addition, longer-term follow-up incorporating broader patient-reported quality-of-life measures will be necessary to determine whether short-term symptom improvement translates into sustained functional and quality-of-life benefits. Additionally, the study did not evaluate whether decompression on the concave versus convex side influenced outcomes. Nonetheless, the clinical and radiological trends observed here may inform power calculations and guide the design of future prospective trials.

To build on these findings, future research should focus on obtaining long-term data to better characterize the durability of clinical and radiological outcomes. A multicenter, cross-campus prospective study is currently underway to evaluate minimally invasive decompression-alone strategies in this population, with extended follow-up to monitor curve progression, reoperation rates, and spinal balance over time. By addressing these aspects, future work will help refine patient selection criteria and clarify the role of minimally invasive decompression in the management of mild spinal deformity.

## 5. Conclusions

This study highlights navigated tULBD as a promising, minimally invasive option for patients with mild ADS and DLSS. The procedure resulted in significant reductions in pain and disability without adversely affecting spinal alignment. These short-term findings indicate that tULBD is effective in addressing symptoms in this complex patient population. However, further research is required to evaluate the long-term durability of these outcomes and to assess its efficacy in patients with more severe spinal deformities.

## Figures and Tables

**Table 1:** Sociodemographic, Clinical, Hospitalization, and Surgical Characteristics of Study Patients.

Variables	Total (N = 20)
Age at Surgery (years), mean ± SD	74.6 ± 7.6
Gender, n (%)	
Male	12 (60)
Female	8 (40)
BMI, kg/m^2^, mean ± SD	26.08 ± 3.7
Weakness, n (%)	
Yes	13 (65)
No	7 (35)
Number of levels Operated, median (IQR)	2 (1–3)
Number of levels Operated, n (%)	
1 Level	9 (45)
2 Levels	5 (25)
3 Levels	5 (25)
5 Levels	1 (5)
Anatomical Level Operated, n (%)	
T11/12	1 (2.6)
L1/2	1 (2.6)
L2/3	9 (23.1)
L3/4	15 (38.5)
L4/5	10 (25.6)
L5/S1	3 (7.6)
Surgery Time, minutes, mean ± SD	130.1 ± 52.7
Blood Loss, mls, mean ± SD	43.3 ± 14.2
Length of Hospital Stay, hours, median (IQR)	29 (24–32.3)
Follow-up, months, median (IQR)	2 (1–6)

SD, Standard Deviation; BMI, body mass index; IQR, interquartile range.

**Table 2:** Radiological Parameters: Pre-Operative vs. Post-Operative Outcomes for Patients in the Study.

Radiological Parameter	Pre-Op	Post-Op	*p*-Value
Pelvic Incidence, median (IQR)	54.8° (46.7–62.3°)	53.8° (47.3–62.3°)	0.07
Pelvic Tilt, median (IQR)	28.9° (22–30.9°)	26.3° (22–32.1°)	0.16
Sacral Slope, median (IQR)	31.4° (24.9–33.3°)	29.4° (26.3–33.8°)	0.70
Lumbar Lordosis, mean ± SD	42.8° ± 15.5°	42.4° ± 14.8°	0.75
Sagittal Vertical Axis, mm, median (IQR)	34.4 (22.4–56.8)	36.2 (15.4–62)	0.53
Global Cobb Angle	18.6° (14.8–23.4°)	19.6° (15.4–26.1°)	0.08
Segmental Cobb Angle (operated level), median (IQR)	6.0° (2.3–10.4°)	5.7° (1.7–9.9°)	0.53
Spondylolisthesis (mm), median (IQR)	−0.32 (−1.16–1.14)	−0.26 (−1.38–2.47)	0.71
Lateral Listhesis (mm), mean ± SD	0.08 ± 3.79	0.24 ± 4.08	0.19

SD, Standard Deviation; BMI, body mass index; IQR, interquartile range; mm, millimeters.

## Data Availability

The data presented here are available upon request from the corresponding author. They are not publicly available because of ethical concerns.

## Abbreviations

The following abbreviations are used in this manuscript:

ADH	Anterior Disk Height
ASA	American Society of Anesthesiologists
DH	Disk Height
LL	Lumbar Lordosis
SL	Segmental Lordosis
MIS	Minimally Invasive Surgery
ULBD	Unilateral Laminotomy for Bilateral Decompression
ODI	Oswestry Disability Index
PDH	Posterior Disk Height
NRS-B	Numeric Rating Scale for Back Pain
NRS-L	Numeric Rating Scale for Leg Pain
CT	Computed Tomography
MRI	Magnetic Resonance Imaging
ADS	Adult Degenerative Scoliosis
DLSS	Degenerative Lumbar Spinal Stenosis
MISS	Minimally Invasive Spine Surgery
CSF	Cerebrospinal Fluid
PI	Pelvic Incidence
PT	Pelvic Tilt
SS	Sagittal Slope
SVA	Sagittal Vertical Axis
GCA	Global Cobb Angle
SCA	Segmental Cobb Angle
